# Design and Pilot Study of a High-Fidelity Medical Simulation of a Hospital-Wide Cybersecurity Attack

**DOI:** 10.21203/rs.3.rs-3959502/v1

**Published:** 2024-04-05

**Authors:** Brennan Marsh-Armstrong, Fernanda Pacheco, Christian Dameff, Jeffrey Tully

**Affiliations:** University of California San Diego School of Medicine; University of California San Diego School of Medicine; University of California San Diego Medical Center; University of California San Diego Medical Center

**Keywords:** cybersecurity, medical education, simulation, healthcare, malware, ransomware

## Abstract

**Background:**

Cybersecurity incidents affecting hospitals have grown in prevalence and consequence over the last two decades, increasing the importance of cybersecurity preparedness and response training to minimize clinical disruptions. This work describes the development, execution, and post-exercise assessment of a novel simulation scenario consisting of four interlocking intensive care unit (ICU) patient scenarios. This simulation was designed to demonstrate the management of acute pathologies without access to conventional treatment methods during a cybersecurity incident in order to raise clinician awareness of the increasing incidence and patient safety implications of such events.

**Methods:**

The simulation was developed by a multidisciplinary team of physicians, simulation experts, and medical education experts at UCSD School of Medicine. The simulation involves the treatment of four patients, respectively experiencing postoperative hemorrhage, end stage renal disease, diabetic ketoacidosis, and hypoxic respiratory failure, all without access to networked medical resources. The simulation was first executed as part of the proceedings of CyberMed Summit, a healthcare cybersecurity conference in La Jolla, California, on November 19th, 2022. Following the simulation, a debrief session was held with the learner in front of conference attendees, with additional questioning and discussion prompted by attendee input.

**Results:**

Though limited to a single subject by the pilot-study nature of this research, the physician learner successfully identified the acute etiologies and managed the patients’ acute decompensations while lacking access to the hospital’s electronic medical records (EMRs), laboratory results, imaging, and communication systems. Review of footage of the event and post-experience interviews yielded numerous insights on the specific physician-focused challenges and possible solutions to a hospital-infrastructure-crippling cyber attack.

**Conclusion:**

Healthcare cybersecurity incidents are known to result in significant disruption of clinical activities and can be viewed through a patient-safety oriented perspective. Simulation training may be a particularly effective method for raising clinician awareness of and preparedness for these events, though further research is required.

## Background

Since the early 2000s, the prevalence, complexity, and consequences of malware attacks on health care organizations have steadily risen. Incomplete reporting makes accurate metrics challenging to obtain, but known malware attacks targeting healthcare groups in the United States have doubled in the last 5 years and tripled in the last decade.^1,2^ There were over 1,400 distinct attacks weekly in 2022, comprising roughly 25% of all recorded US-facing cyberattacks.^3^ Healthcare is among the most threatened industrial sectors in the US.^3^ While healthcare is highly targeted, metrics suggest that the sector is not sufficiently protected. Each year, over 500 major breaches occur, compromising the personal health data of 40 to 110 million Americans.^4,5^

The consequences of these attacks are extensive and multifaceted. The data obtained from the aforementioned breaches result in approximately 5 million cases of identity theft annually, resulting in an average of $10,000 of unrecoverable losses per victim.^6^ A data breach costs a hospital itself 15 million dollars on average.^5^ Other serious consequences include reduced hospital activity, missed appointments, loss of patient trust, and worse patient health outcomes.^7,8^

Medical systems are frequently targeted by hackers because of their extensive quantities of valuable personal health information, insufficient investment in cybersecurity measures, and vast attack surfaces secondary to biotechnical infrastructure’s increasing interconnectedness.^8^ In recent years, the reported incidence of large-scale hospital ransomware attacks have multiplied. The Hollywood Presbyterian ransomware attack in 2016 was the first highly publicized case of such incidents.^9^ The 2017 “WannaCry” international ransomware attack is arguably the most infamous episode affecting healthcare delivery organizations, costing the National Health Service (NHS) in the United Kingdom over $100 million dollars, and subsequent incidents have reported even greater financial losses.^10^ The number of successful cyberattacks further increased globally as a result of the greater reliance on network and cloud resources required by the COVID-19 pandemic.^11^

Many healthcare delivery organizations train staff on how to reduce their susceptibility to cyberattacks, particularly phishing.^12^ While didactic education modules for clinical staff have been developed, no training modality to-date has been established as the standard for educating staff or been proven to reduce likelihood of cybersecurity incidents.^13–15^ As the increasing patient care impacts of cybersecurity incidents are emphasized, training, not just for the prevention of but the response to and mitigation of impact from cybersecurity incidents, may be important for the practicing clinician to minimize patient harm.

Simulation training is a cornerstone of medical education.^16^ Accredited US medical training programs employ simulations to practice emergency room, labor and delivery, intensive care unit (ICU), trauma bay, and hospital-wide disaster scenarios.^17,18^ Simulation training is unique as a learning modality as it facilitates practice of high-risk scenarios with high-fidelity without exposure to unwanted consequences. These traits make simulation training ideal for teaching medical personnel how to respond to the clinical impacts of cybersecurity incidents. Prior studies detail simulations designed to teach physicians how to identify hacked medical devices in patient presentations and recognize when medical lab values were being maliciously manipulated have been described.^19,20^ We report the development of a novel high-fidelity clinical simulation of a hospital-wide networked-resource disruption in which physicians needed to adapt their care for multiple acutely ill ICU patients. We additionally describe physician and observer feedback from the first execution of the simulation.

## Methods

### Simulation development

We reviewed acute pathologies commonly treated in ICUs and identified four which met the following criteria: 1) decompensation occurs in a time sensitive fashion with inadequate treatment, 2) conventional management guidelines rely in part on medical imaging, labs, EMRs, or intrahospital communication tools, and 3) effective management can be provided via alternative methods in a disaster scenario. After a clinical discussion between multidisciplinary physicians and simulation/medical education experts, postoperative bleeding, hypokalemia secondary to treatment for diabetic ketoacidosis (DKA), volume overload secondary to end stage renal disease (ESRD), and COVID-19 induced acute hypoxemic respiratory failure were selected. A scenario was constructed in which four patients with the aforementioned pathologies would require simultaneous management. The simulation aimed to realistically replicate the multitasking required of an ICU physician, while providing opportunities to emphasize the challenge of providing critical care in the absence of technology. The individual patient cases and their treatment outcomes were written independently before being integrated into a single simulated ICU case.

The full simulation scenario, including specific vitals, lab values, and predicted decision-paths is available as Supplemental 1. Individual cases are summarized below.

### Case 1:

Case 1 features a patient with postoperative hemorrhage requiring a workup subsequently hampered by lack of access to diagnostic imaging, labs, or inter-team communication. The patient, represented by a simulation mannequin, is a 54-year-old male with a past medical history of diverticulosis who presented to the hospital the day prior for a colonic perforation secondary to suspected acute diverticulitis and is now recovering in the ICU after a partial colectomy complicated by significant blood loss. On initial presentation, he will have borderline hypotensive blood pressure and abdominal pain not fully controlled by the scheduled morphine and hydromorphone. The goals of this first encounter aim for the physician to perform an effective interview and physical exam, optimize pain management, and direct someone to carefully monitor vitals, before leaving the room to examine other patients. When the physician next returns, the patient will have worsening pain, mental status, and vitals indicative of mild shock. Abdominal ultrasound at this time will demonstrate intra-abdominal bleeding. All other imaging will be unavailable due to a cybersecurity incident affecting the PACS system and related programs. If a pRBC cross match is requested the electronic blood bank, management system will also be unavailable. Intrahospital communication systems like Voice over IP (VOIP) phones will not work as a means of reaching other teams. The learning goals for the second encounter of the first case will be to recognize the need for and then perform a bedside ultrasound, request and administer uncrossed O- pRBCs, establish communication with the surgery team, and discuss with the surgeon the risks and benefits of performing an exploratory laparotomy on the patient despite a lack of clinical imaging. The surgeon will arrive at the bedside and decide to proceed to the operating room.

### Case 2:

The second case presents an ESRD patient with volume overload requiring dialysis without access to the patient records detailing the patient’s historical dialysis protocol. It also requires the physician to explain a network outage to a concerned patient. The patient is a 55-year-old male, played by a standardized patient, with ESRD on dialysis who was admitted for volume overload and hyperkalemia. On examination and ED labs respectively, he has signs of fluid overload and metabolic acidosis. He missed his last dialysis appointment and is currently awaiting inpatient dialysis. During the examination, there will be an overhead announcement reporting the hospital network downtime. After the announcement the patient will be concerned and ask the physician the significance of the downtime. The physician’s learning goals are to share relevant information regarding the downtime with the patient in an appropriate manner before initiating a standard dialysis protocol with added low-dose potassium (appropriate for most dialysis patients in an acute context). With these goals accomplished, the physician will conclude their interaction with the patient.

### Case 3:

Case 3 features the workup of diabetic ketoacidosis and subsequent hypokalemia. Patient 3 is a 32-year-old male admitted to the ICU an hour prior from the ED for DKA, presenting with abdominal pain, nausea, fatigue, stiffness (as a subtle sign of hypokalemia), sunken eyes, and tachycardia. His electrocardiogram (ECG) is notable for rare premature ventricular contractions (PVCs) without U waves. A point of care glucose will show moderate DKA-range hyperglycemia. All other pertinent labs including serum potassium will be unavailable. The goal of the first encounter will be to recognize the patient has DKA and initiate treatment with fluid repletion and insulin, monitored only with regular point of care glucose labs. When the physician next returns, the patient will have become obtunded, unresponsive, and hypoxic, with non-specific arrhythmic telemetry. ECG will show torsades de pointes and the nurse will report he is pulseless. The goal of the second encounter will be to initiate a code, provide ACLS, identify the causes as hypokalemia and hypomagnesemia, and administer potassium and magnesium, at which point spontaneous circulation will return.

### Case 4:

The fourth case simulates management of hypoxemic respiratory failure secondary to COVID-19 pneumonia. Patient 4 is a 63-year-old female who recently presented with fatigue, shortness of breath, and cough to the ED and was thereafter found to have COVID pneumonia. Her respiratory status has been declining for the last day, is now requiring oxygen via high-flow nasal cannula, and has already been consented for possible intubation. Given the paging system downtime, the patient’s nurse will have been unable to find another physician capable of intubation and will interrupt the physician from their previous case to assist them. The goal of the second encounter will be to recognize the risks inherent to a downed communication network in a hospital and perform a rapid sequence intubation of the patient.

### Execution of the simulation:

The initial execution of this simulation took place at the UC San Diego School of Medicine Simulation Training Center in La Jolla, California on November 11th, 2022 as part of the content of CyberMed Summit, a multidisciplinary healthcare cybersecurity conference.

Patients 1, 2, and 3 were situated within separate stalls in the center’s ICU room. Patient 1 was a manikin on a hospital bed with abdominal postoperative dressing, patient 2 was a standardized patient in street clothing on a hospital bed, and patient 3 was a manikin in a hospital gown on a hospital bed. Manikins were high-fidelity Laerdal SimMan 3Gs (Laerdal, Stavanger, Norway), and voice acting was provided by standardized patients in an adjacent control room. Patient 4 was also a Laerdal SimMan 3G manikin voiced by a standardized patient, but was placed in an adjacent hospital-like simulation room. The simulation was managed by a simulation professional in a control room between the two simulation rooms in use. In addition to the confederate nurse, additional personnel included the clinician who provided the initial handoff of patients, a second nurse who provided communication about Patient 4 and remained to assist with additional tasks, and the surgeon who appeared to take Patient 1 to the operating room.

After receiving verbal sign-out on the first three cases from a physician colleague, the learner was asked by a confederate nurse to begin evaluation of Patient 1. After initiating treatment for Patient 1, the learner was directed to begin the encounter with Patient 2. Halfway through this encounter an overhead announcement indicated indefinite network downtime and limited electronic resource availability in the hospital. The concerned Patient 2 prompted the learner to explain the situation and its potential impacts on the patient’s care. After this conversation, the learner was prompted to move to Patient 3. Upon completion of the first portion of this encounter, another confederate nurse entered the room, explained Patient 4’s situation, and led the learner out of the room to conduct the Patient 4 encounter in the adjacent hospital room. While the learner was out of the room, the simulation runner saturated Patient 1’s surgical dressings with fake blood. When the learner returned to the ICU room, they found Patient 1 dangerously hypotensive and in acute distress. The nurse called the blood bank and was informed that they are unable to perform crossmatches. The patient’s surgeon arrived so that the learner can convince them to return to the hospital to repair a suspected postoperative bleed, per above. As Patient 1 is rolled back, Patient 3’s vital sign monitor started alarming, leading into the second half of the Patient 3 encounter. Once several cycles of ACLS are performed, Patient 3’s vitals were stabilized and the simulation concluded.

After completing the simulation, the learner debriefed with a simulation professional to reinforce the lessons they learned. This session took place in front of conference attendees who were able to ask the learner and simulation professional additional questions in an expanded discussion.

## Results

The first learner to experience this simulation, a pulmonary critical care trained intensivist, successfully completed the exercise with minimal redirecting required by the confederate nurse. There were no technical or logistical errors with simulation flow or execution. Both video and audio were recorded for the simulation and debrief. Video recordings and full transcripts of both are included as Supplemental 2.

While the learner did not immediately identify a potential cybersecurity incident as the result of the downtime of the EMR, imaging, and other technical systems, they did have prior real-world experience with practicing clinically during a ransomware incident, experience which they relied upon in communicating with the distressed Patient 2. “You just have to be there to advocate for your patient,” they responded, when asked during the debrief about their approach to the patient’s anxious inquiries. The standardized patient, also a participant in the debrief, voiced agreement. “What gives you reassurance as a patient is when everything seems to be going at a normal cadence,” they explained, “and the moment [the patient] sense(s) that there is a break in the cadence- especially for an urgent, care related issue- it causes concern…one of the ways we try to handle it is being honest, in which [the learner] acknowledged there was an issue, and that [patient care] was being addressed.” The dynamic, interconnected multi-patient nature of the simulation resonated in a positive way with the learner. “Most of our [historical] simulations are just about one patient, so it is a bit different juggling four of them, but I guess that’s more of what we do in a normal ICU, so it’s a good simulation from that standpoint.”

## Discussion

Modern clinical practice is increasingly dependent on the use of technology at the bedside. Medical education has correspondingly placed significant emphasis on training learners to integrate the use of medical devices, electronic medical records, patient portals, and other technologic systems alongside classical instruction in the physical examination and differential diagnosis. This focus on producing technically literate clinicians has not yet widely encompassed cybersecurity elements, despite the increasing operational and clinical impact of the topic on our healthcare system.

Simulation has become a foundational component of medical education and is particularly suited for training response to infrequent, high stakes clinical situations in which preparedness may prevent significant morbidity and mortality. Simulation exercises for clinical cybersecurity incidents involving vulnerable medical devices have previously been reported, but, to our knowledge, this report details the first clinical cybersecurity simulation depicting the potential patient safety consequences of a ransomware attack.

The literature contains little evidence or data concerning the clinical impact of ransomware, but media coverage, trade association surveys, and government reports indicate the potential for significant disruption or degradation of clinical care. Though examples of alleged morbidity and mortality secondary to inaccessible clinical monitoring, absence of clinical decision support software, and discontinuity of care provide the foundation for potential scenarios, an approach focusing on the standard of care for time-sensitive, critical medical conditions then disrupted by the unavailability of technologically dependent components of those standards may be equally useful in training clinical cybersecurity preparedness and resiliency.

We thus developed four scenarios in which acute pathologies required management without access to standard technologically dependent medical interventions in an attempt to recreate conditions which may be present during a severe ransomware attack in which multiple connected systems may be down. The study employed standardized patients along with high-fidelity manikins to simulate acute patient encounters in an ICU setting. The learner utilized their prior knowledge and skills to successfully manage acute issues with all four patients.

The primary goal of this simulation was to assess the learner’s clinical management of acutely ill patients in the midst of hospital-wide system downtimes. While the ability to adapt to a degraded environment is a key component of reducing risk of morbidity or mortality in a disaster scenario, future iterations or separate exercises could explore incorporating the deployment of cybersecurity specific emergency responses. Institution-wide, cybersecurity-specific emergency operations plans developed and run by emergency management departments are recommended by several industry guidelines. Incorporating clinical simulation exercises such as this scenario alongside table top exercises, downtime drills, and regional coordination may be an effective process for iterative development of comprehensive cybersecurity incident response plans. Though, further repeats of this simulation are required for generalizable conclusions about simulation’s effectiveness at teaching cybersecurity-attack preparedness to physicians.

This report has several limitations. Most notably, as a pilot study with a single subject, the generalizability of this study’s findings lack statistical power. That said, as a proof of concept it both confirms the feasibility of such a simulation and it’s potential capacity for effective physician education. Further, access to a high-fidelity simulation center staffed by experienced professionals is a luxury that many institutions and healthcare delivery organizations may not possess. We describe the results of the exercise after a single run-through with the simulation creators, who have previously executed clinical cybersecurity simulations. Subsequent episodes may uncover challenges or technical issues that render it less generalizable or implementable. Further medical education research focusing both on how cybersecurity training may be most effectively integrated into curricula and how such training may improve real world preparedness and response is warranted.

We describe the development of a clinical cybersecurity simulation focused on the management of critically ill patients during downtime resulting from a ransomware attack. The first execution of the simulation saw the learner successfully navigating clinical challenges arising from the unavailability of critical laboratory, imaging, and record systems. Such exercises may be a helpful way to prepare clinicians to respond to cybersecurity incidents. Further research of both this medical simulation and high-fidelity medical simulations in general is required to elucidate their effectiveness at teaching cyberattack readiness to physician.

## Data Availability

The dataset supporting the conclusions of this article, as well as a full design document for the studied simulation, is included within the article as supplemental files.

## Abbreviations

ICU: Intensive care unit
UCSD: University of California, San Diego
EMR: Electronic medical records
NHS: National Health Service
ICU: Intensive care unit
ESRD: End-stage renal disease
DKA: Diabetic ketoacidosis
VOIP: Voice over IP
pRBC: Packed red blood cells
ED: Emergency department
ECG: Electrocardiogram
PVC: Premature ventricular contractions
ACLS: Advanced cardiac life support
COVID-19: Coronavirus disease of 2019
